# Short-term consequences of daily consumption of the quinoa (*Chenopodium quinoa* Willd.) diet in normal and diabetic rats

**DOI:** 10.14202/vetworld.2025.1715-1724

**Published:** 2025-06-26

**Authors:** Murali Adiga, S. D. Manjula, Dhiren Punja, Chakravarthy Marx Sadacharan, Dinesh Upadhya, K. Vasanthalaxmi, Nanda Acharya, Chinmay Suryavanshi

**Affiliations:** 1 Department of Physiology, Kasturba Medical College, Manipal, Manipal Academy of Higher Education, Manipal, India; 2 Department of Biomedical Sciences, Tilman J. Fertitta Family College of Medicine, University of Houston, Texas, USA; 3 Center for Molecular Neurosciences, Kasturba Medical College, Manipal, Manipal Academy of Higher Education, Manipal, India

**Keywords:** diabetes mellitus, dietary intervention, glycemic control, lipid profile, liver function, quinoa, rat model, streptozotocin

## Abstract

**Background and Aim:**

*Chenopodium quinoa* Willd. (Quinoa) is a nutrient-dense pseudocereal with potential therapeutic benefits for metabolic disorders, including diabetes mellitus. However, the safety and efficacy of varying concentrations of dietary quinoa on metabolic and histological parameters in diabetic and non-diabetic models remain underexplored. This study aimed to evaluate the short-term effects of different quinoa supplementation levels (0%, 20%, 40%, and 80%) on glycemic control, lipid metabolism, hepatic and renal function, hematological indices, and organ histopathology in normal and streptozotocin (STZ)-induced diabetic rats.

**Materials and Methods:**

Forty-eight adult male Wistar rats were randomly assigned to eight groups (n = 6 each) based on diabetic status and dietary quinoa concentration. Diabetes was induced using low-dose STZ (25 mg/kg). Animals received the respective quinoa-enriched diets for 30 days. Blood glucose, glycated hemoglobin, lipid profiles, liver/kidney function markers, and complete blood counts were analyzed. Histological assessments of liver and kidney tissues were also performed.

**Results:**

Diabetic rats receiving 40% and 80% quinoa diets exhibited significant reductions in fasting blood glucose (p < 0.05) and alanine transaminase levels (p < 0.01), indicating improved glycemic and hepatic function. Very low-density lipoprotein cholesterol decreased significantly in all quinoa-fed diabetic groups, and high-density lipoprotein cholesterol increased notably in the 20% quinoa group (p < 0.05). Normal rats showed no adverse changes across biochemical or hematological indices. Histological analysis confirmed the absence of morphological abnormalities in hepatic and renal tissues in all groups.

**Conclusion:**

Short-term dietary quinoa supplementation, particularly at 40% and 80% inclusion levels, effectively improves glycemic and lipid profiles and mitigates liver enzyme elevations in diabetic rats without compromising health parameters in normal controls. The findings support quinoa’s potential as a safe dietary adjunct in managing diabetes-related metabolic dysfunctions.

## INTRODUCTION

Diabetes mellitus (DM) represents a significant global health concern, currently affecting an estimated 537 million adults worldwide. Chronic hyperglyce-mia associated with DM contributes to numer-ous complications, including diabetic retinopathy, neuropathy, nephropathy, and cognitive decline [1–3]. These outcomes underscore the pressing need for accessible and effective therapeutic strategies. While pharmacological treatments remain foundational in DM management, dietary interventions are increasingly recognized for their ability to improve metabolic parameters with minimal adverse effects.

*Chenopodium quinoa* Willd. (Quinoa), a pseudocereal indigenous to Latin America, has garnered attention as a potential dietary intervention for metabolic disorders. It possesses a favorable nutritional profile, comprising 12%–16% high-quality protein with a complete set of essential amino acids [4], approximately 8.9% dietary fiber [5], and a rich array of phytochemicals, including phytosterols, carotenoids, phenolics, and betacyanins [6, 7]. Furthermore, its low-to-moderate glycemic index (35–53, depending on preparation methods) [4] and the presence of bioactive compounds such as flavonoids, squalene, and saponins may contribute to its beneficial metabolic effects [8, 9].

Preclinical studies have highlighted quinoa’s diverse therapeutic properties. In high-fructosefed rats, quinoa consumption improved systemic oxidative status, as evidenced by decreased plasma malondialdehyde levels and enhanced antioxidant defenses, including increased plasma glutathione peroxidase, catalase, and erythrocyte superoxide dismutase [10]. Additional studies have reported quinoa’s efficacy in ameliorating conditions such as obesity, diabetes, and prediabetes. In murine models, quinoa intake significantly reduced plasma total cholesterol and low-density lipoprotein (LDL) cholesterol and attenuated hepatic steatosis relative to control groups [11]. Moreover, investigations into quinoa flour and protein isolates have characterized their antinutritional factors, physicochemical properties, and rheological properties, as well as their antioxidant capacity [12–14].

Clinical evidence further supports quinoa’s potential benefits. Consumption of quinoa has been associated with reduced glycated hemoglobin (HbA1c) levels and body mass index in individuals with prediabetes [15]. Favorable changes in lipid parameters, including reductions in triglycerides and cholesterol, have also been reported [16, 17]. The high protein and fiber content of quinoa may play a key role in mediating these effects, positioning it as a promising dietary adjunct for managing obesity and DM [18].

Despite accumulating evidence highlighting quinoa’s beneficial effects on metabolic health, several critical knowledge gaps persist. Most preclinical studies have employed high-fat diet or fructose-induced models of metabolic dysfunction, with limited investigations utilizing low-dose streptozotocin (STZ)-induced diabetic models that more closely mimic the pathophysiology of type 2 diabetes. Furthermore, while quinoa’s biochemical composition suggests therapeutic potential, few studies have systematically evaluated its dose-dependent effects on glycemic regulation, lipid metabolism, hepatic and renal function, and histopathological outcomes in both diabetic and non-diabetic settings. In addition, the short-term safety profile of high-percentage quinoa diets remains insufficiently characterized, particularly in non-diabetic models, which is essential for determining its translational applicability.

In light of these gaps, the present study aimed to investigate the short-term effects of dietary quinoa supplementation at graded concentrations (0%, 20%, 40%, and 80%) on metabolic, biochemical, hematological, and histopathological parameters in normal and STZ-induced diabetic Wistar rats. Specifically, this study sought to (i) evaluate the efficacy of quinoa in modulating glycemic and lipid profiles, (ii) assess hepatic and renal function through enzymatic and histological analyses, (iii) determine the hematological safety across different diet groups, and (iv) identify the optimal quinoa concentration that confers therapeutic benefit without inducing adverse effects. By addressing these objectives, the study provides a robust foundation for future translational research on quinoa-based dietary interventions for metabolic disorders.

## MATERIALS AND METHODS

### Ethical approval

All experimental procedures were conducted following ethical approval from the Institutional Animal Ethics Committee (IAEC/KMC/36/2022, dated March 26, 2022). The study adhered to the ARRIVE guidelines for animal care and euthanasia.

### Study period and location

The study was conducted from January 1, 2023 to January 30, 2023 at the Central Animal Research Facility, Manipal Academy of Higher Education.

### Animal model

Adult male Wistar rats (10–12 months old), weighing between 250 g and 300 g, were used in this study. The rats were maintained under standard laboratory conditions at 22°C with a 12-h light/dark cycle and had *ad libitum* access to standard rat chow and water. Two animals were housed per cage throughout the study. Food intake was recorded daily to monitor dietary consumption across groups.

### Experimental design

#### Group allocation

Forty-eight rats were randomly assigned to eight experimental groups (n = 6 per group) using a computer-generated randomization protocol. The animals were categorized based on diabetic status and the percentage of dietary quinoa supplementation:

- Normal rats (N):
  - N0: Control (0% quinoa)
  - N20: 20% quinoa diet
  - N40: 40% quinoa diet
  - N80: 80% quinoa diet

- Diabetic rats (D):
  - D0: Diabetic control (0% quinoa)
  - D20: 20% quinoa diet
  - D40: 40% quinoa diet
  - D80: 80% quinoa diet.

#### Quinoa diet preparation

Commercial quinoa powder (Nature Vit, India) containing 16% protein and 8% dietary fiber was blended with standard rat chow in designated ratios to create the test diets:

- 0% diet: Standard chow only
- 20% diet: 20% quinoa flour + 80% chow
- 40% diet: 40% quinoa flour + 60% chow
- 80% diet: 80% quinoa flour + 20% chow.

Pellets were formed by mixing the respective formulations with boiling water, followed by sun drying and storage at room temperature (20°C–25°C). All groups were provided their respective diets *ad libitum*.

### Induction of DM

DM was induced through a single intraperitoneal injection of STZ at a dose of 25 mg/kg body weight (Cayman Chemicals, USA; Catalog No. 13104) following overnight fasting, as described previously by Guo *et al*. [19]. Blood glucose levels were measured at 72 h and again after 1 week to confirm hyperglycemia (>200 mg/dL). To prevent hypoglycemia, 10% sucrose solution was administered for 1 week post-STZ injection.

### Sample collection and processing

After 30 days of dietary intervention, blood samples were collected through direct cardiac puncture under deep anesthesia into appropriately labeled tubes. Samples were allowed to clot at 20°C–25°C for 30 min and centrifuged at 4000 × *g* for 5 min at 4°C. The resulting serum was aliquoted and stored at −80°C until biochemical analyses were performed.

### Hematological and biochemical analysis

**Figure 1:**
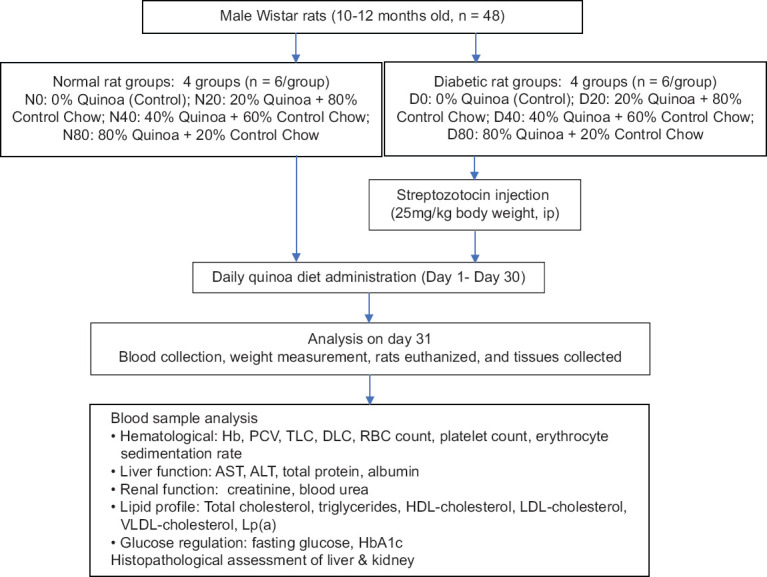
Methodology summary.

Serum was analyzed for aspartate transaminase (AST), alanine transaminase (ALT), total protein, albumin, urea, creatinine, total cholesterol, triglycerides, LDL cholesterol, high-density lipoprotein (HDL) cholesterol, HbA1c, and fasting blood glucose levels (summarized in Figure 1). Hematological parameters were assessed using blood collected in ethylenediaminetetraacetic acid-coated vacutainers (BD Vacutainer, USA). All assays were conducted using automated analyzers (Star-20 clinical chemistry analyzer, India, and Nihon Kohden hematology analyzer, Japan) at the Central Animal Research Facility.

### Histological examination

At the end of the experimental period, the rats were euthanized under deep anesthesia. Perfusion fixation was performed transcardially using 4% paraformaldehyde (Sigma-Aldrich, USA) in phosphate-buffered saline (pH 7.4). Liver and kidney tissues were harvested, fixed in 10% neutral buffered formalin for 48–72 h, and processed through standard histol-ogical procedures. Tissues were dehydrated, cleared in xylene, embedded in paraffin, and sectioned at 5 μm thickness using a rotary microtome (Leica RM 2155, Germany). Hematoxylin and eosin staining was performed, and sections were examined using a widefield microscope (Motic Red 200, China) with digital image capture and analysis through Motic Image-Plus 2.0 software.

### Statistical analysis

Data were analyzed using GraphPad Prism version 9.0 (GraphPad Software, USA). The Shapiro–Wilk test was employed to assess data normality, while Levene’s test evaluated the homogeneity of variances. Group comparisons across different quinoa concentrations (0%, 20%, 40%, and 80%) were conducted using a one-way analysis of variance (ANOVA), followed by Newman-Keuls multiple comparison *post hoc* testing. Body weight changes over the study period were analyzed using two-way ANOVA. Results are presented as mean ± standard deviation (SD), with p < 0.05 considered statistically significant.

## RESULTS

### Impact of quinoa supplementation on body weight and glycemic control

#### Body weight

**Figure 2:**
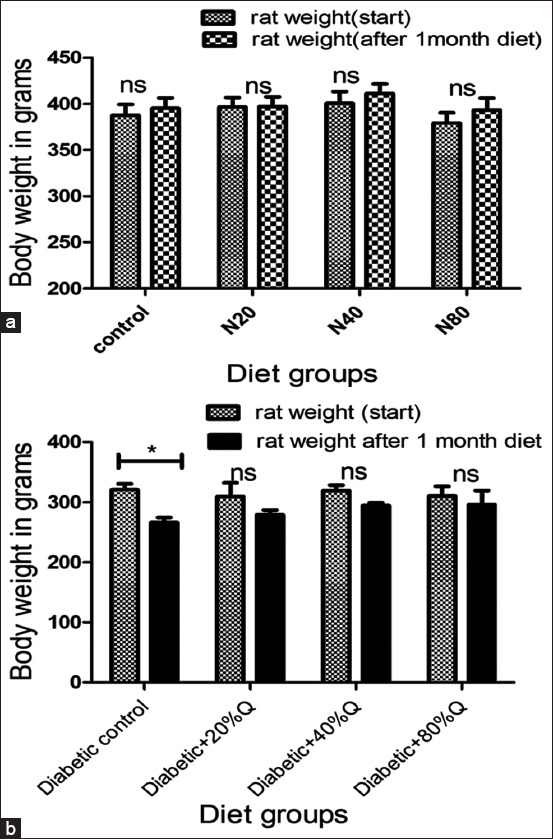
Body weight changes in rats after 1 month of quinoa supplementation. (a) Normal rats and (b) diabetic rats fed 0%–80% quinoa diets. *p < 0.05; ns=Not significant.

Figure 2 presents the changes in body weight among normal and diabetic rats over the 30-day dietary intervention period. All normal rat groups (N0, N20, N40, and N80) exhibited an increase in body weight (Figure 2a); however, these gains were not statistically significant. Conversely, diabetic rats demonstrated variable weight loss across groups, with the D0, D20, D40, and D80 groups showing reductions of 17%, 9.7%, 7.8%, and 4.5%, respectively (Figure 2b). Notably, only the diabetic control group (D0) exhibited a statistically significant reduction in body weight (17%, p < 0.05).

#### Fasting blood glucose and HbA1c

Fasting blood glucose levels in normal rats remained stable within the range of 74–89 mg/dL throughout the study. In contrast, diabetic rats exhibited markedly elevated glucose levels (242–293 mg/dL). A downward trend in fasting glucose was observed in quinoa-supplemented diabetic groups, with the D40 and D80 groups showing statistically significant reductions (17%, p < 0.05) compared to the diabetic control (243 ± 15 and 242 ± 30 vs. 293 ± 42 mg/dL). Although a reduction was observed in the D20 group, the change did not reach statistical significance. HbA1c levels remained consistent in normal rats but were elevated in all diabetic groups (Table 1).

**Table 1:** Biochemical parameters in normal and diabetic rats.

Parameters	Normal rats, mean (SD)				Diabetic rats, mean (SD)			
	
	N0	N20	N40	N80	D0	D20	D40	D80
Fasting glucose (mg/dL)	74 (6)	89 (6)	82 (8)	87 (4)	293 (42)	281 (28)	243 (15)*	242 (30)*
HbA1C (%)	3.65 (0.27)	3.72 (0.2)	3.6 (0.2)	3.7 (0.3)	5.4 (0.2)	5.2 (0.1)	4.9 (0.2)	4.8 (0.2)
AST (U/L)	117 (5)	127 (6)	112 (2)	115 (5)	126 (7.2)	133 (11)	120 (7)	122 (6)
ALT (U/L)	57 (4)	51 (3)	54 (5)	53 (7)	89 (17)	66 (3)**	68 (6)**	67 (8)**
Total proteins (g/dL)	7.3 (0.1)	7.5 (0.2)	7.4 (0.2)	7.2 (0.2)	6.9 (0.4)	7.2 (1.2)	6.9 (0.3)	7 (0.4)
Albumin (g/dL)	3.2 (0.1)	3.3 (0.2)	3.2 (0.1)	3.4 (0.3)	2.9 (0.2)	2.5 (0.2)	2.7 (0.2)	2.6 (0.3)
Urea (mg/dL)	18 (1)	22 (2)	20 (0.8)	19 (0.6)	23 (6)	31 (12)	30 (6)	31 (7)
Creatinine (mg/dL)	0.7 (0.1)	0.7 (0.1)	0.76 (0.1)	0.7 (0.1)	0.7 (0.1)	0.8 (0.1)	0.7 (0.0)	0.7 (0.1)
Total cholesterol (mg/dL)	83 (4)	77 (1)	74 (2)	73 (2)	133 (7)	133 (8)	142 (8)	140 (10)
Triglycerides (mg/dL)	72 (6)	59 (8)	64 (4)	66 (5)	165 (33)	151 (38)	160 (66)	162 (25)
HDL-C (mg/dL)	36 (2)	37 (3)	38 (4)	40 (5)	35 (7.5)	43 (2)*	40 (3)	38 (4)
LDL-C (mg/dL)	42 (3)	39 (2)	38 (2)	37 (3)	65 (5)	66 (7)	79 (31)	67 (10)
VLDL-C (mg/dL)	33 (4)	30 (2)	32 (3)	34 (3)	37 (10)	26 (2)**	30 (3)*	29 (2)*
Lp (a) (mg/dL)	3.3 (0.2)	3.3 (0.1)	3.7 (0.3)	3 (0.2)	6.4 (0.5)	6.7 (0.2)	6.2 (0.6)	6.5 (0.4)

Values are mean±SD (n = 6) (Calculated using one-way ANOVA and Newman-Keuls *post hoc* test). ANOVA=Analysis of variance, HbA1C=Glycated hemoglobin, SD=Standard deviation, AST=Aspartate transaminase, ALT=Alanine transaminase, N0=Normal control, D0=Diabetic control, HDL-C=High-density lipoprotein cholesterol, LDL-C=Low-density lipoprotein cholesterol, VLDL-C=Very low-density lipoprotein cholesterol, Lp (a)=Lipoprotein (a),

* p < 0.05,

** p < 0.01

### Impact on liver and renal function

ALT levels were significantly reduced in all quinoa-supplemented diabetic groups – D20: 66 ± 3, D40: 68 ± 6, and D80: 67 ± 8 U/L – when compared with the diabetic control group (89 ± 17 U/L, p < 0.01). Other hepatic function markers, including AST, total protein, and albumin, did not differ significantly across groups (Table 1). Furthermore, renal function markers such as urea and creatinine remained within normal physiological ranges in all groups, with no statistically significant differences between control and treatment cohorts.

#### Impact on lipid profiles

Very LDL (VLDL) cholesterol levels decreased significantly in all quinoa-fed diabetic groups – D20, D40, and D80 – compared with the diabetic control (26 ± 2, 30 ± 3, and 29 ± 2 vs 37 ± 10 mg/dL, respectively; p < 0.05). In addition, HDL cholesterol levels were significantly elevated in the D20 group (43 ± 2 vs 35 ± 7.5 mg/dL, p < 0.05). No significant changes in lipid parameters were observed in any of the normal (non-diabetic) groups (Table 1).

#### Impact on hematological parameters

No statistically significant differences were observed in hematological parameters among either normal or diabetic rats, regardless of quinoa supplementation levels (Table 2). All hematological indices remained within reference ranges, suggesting the absence of hematotoxic effects.

**Table 2:** Hematological parameters in normal and diabetic rats.

Parameters	Normal rats, mean (SD)				Diabetic rats, mean (SD)			
	
	N0	N20	N40	N80	D0	D20	D40	D80
Hb (g/dL)	14.6 (0.5)	14.9 (0.6)	14.3 (0.4)	14 (0.5)	14.7 (0.5)	14 (1)	13.7 (1.1)	14 (0.8)
PCV (%)	40 (1.7)	40 (2)	39 (0.7)	38 (1.4)	45 (3)	44 (4)	41 (3)	42 (2.5)
RBC count (millions/mm^3^)	8.5 (0.4)	8.7 (0.4)	8.2 (0.1)	8.3 (0.3)	8.2 (0.5)	7.8 (1)	7.5 (0.9)	8 (0.9)
WBC count (thousands/mm^3^)	12.8 (2)	10.7 (1.3)	10.8 (1)	11 (3.8)	9 (2)	10.4 (1.6)	10.2 (1.5)	8 (1.2)
ESR (mm/h)	2 (1)	2 (1)	3 (2)	1 (1)	2 (1)	2 (1)	2 (1)	2 (1)
Platelet count (lakhs/mm^3^)	7 (0.4)	7.3 (0.5)	7.2 (1.2)	7.6 (0.7)	5.6 (0.7)	7.2 (1.4)	6 (0.9)	6 (1)
DLC-neutrophils (%)	2 (1)	4 (2)	2 (1)	3 (1)	2 (1)	2 (1)	2 (1)	2 (1)
DLC-lymphocytes (%)	92 (3)	90 (2)	95 (2)	92 (2)	93 (2)	93 (3)	94 (1)	94 (2)
DLC-monocytes (%)	1 (0)	2 (1)	1 (1)	1 (1)	3 (1)	2 (1)	2 (1)	2 (0)

SD=Standard deviation, Hb=Hemoglobin, PCV=Packed cell volume, ESR=Erythrocyte sedimentation rate, DLC=Differential leukocyte count

#### Histopathological findings

**Figure 3:**
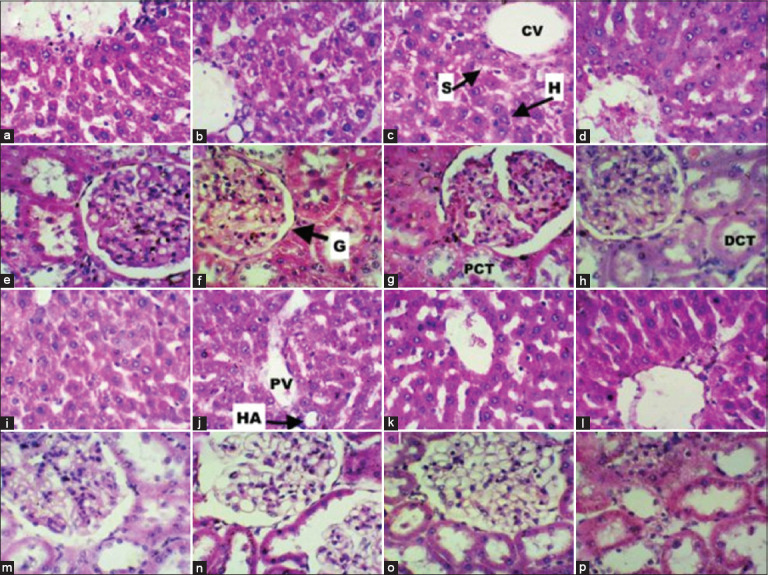
Hematoxylin and eosin-stained liver and kidney sections (40×) from normal and diabetic rats fed varying quinoa diets. Normal rats’ liver (a-d) and kidney (e-h) sections with 0%, 20%, 40%, and 80% quinoa diets, respectively. Diabetic rats’ liver (i-l) and kidney (m-p) sections with 0%, 20%, 40%, and 80% quinoa diets, respectively. PV=Portal vein, S=Sinusoids, H=Hepatocytes, CV=Central vein, HA=Hepatic artery, G=Glomerulus, PCT=Proximal convoluted tubule, DCT=Distal convoluted tubule.

Histological analysis of liver and kidney tissues confirmed the absence of pathological changes across all experimental groups. The liver sections exhibited preserved architecture with intact central veins, normal hepatocytes, and sinusoidal structures. Similarly, the kidneys displayed well-maintained cortical and medullary structures. These findings were consistent across both normal and diabetic rats, irrespective of the quinoa concentration in their diets (Figure 3).

## DISCUSSION

### Therapeutic potential of quinoa in diabetes management

This study provides comprehensive evidence supporting the therapeutic utility of quinoa in the management of diabetes. Significant improvements in glycemic regulation, lipid metabolism, and hepatic function were observed, particularly under diabetic conditions.

### Effects on body weight

The impact of quinoa supplementation on body weight appears to be modulated by several variables, including dietary composition, duration of intervention, and the metabolic state of the subjects. In our study, normal rats receiving quinoa-enriched diets for 30 days did not exhibit significant body weight changes (p > 0.05), in agreement with earlier findings [20, 21]. However, prior research has reported contradictory outcomes. For instance, hydrolyzed quinoa reduced weight gain in sedentary Wistar rats [22], and a high-carbohydrate diet supplemented with fermented or sprouted quinoa decreased food intake and weight gain over 47 days [23].

These discrepancies may stem from differences in saponin content among quinoa varieties and processing techniques, which can lead to loss of saponin fractions during diet preparation [24]. Saponins are known to affect weight regulation by exerting membranolytic activity on intestinal mucosa, inducing food aversion and reduced intake [25]. Although commercially available quinoa undergoes processing to remove bitter-tasting saponins [26], other components such as proteins and dietary fibers may also enhance satiety and reduce caloric intake [27].

In diabetic rats, quinoa supplementation had a protective effect against weight loss. The diabetic control group (D0) experienced a 17% reduction in body weight, whereas supplementation with 20%, 40%, and 80% quinoa resulted in weight loss of 9.7%, 7.8%, and 4.5%, respectively. These findings suggest that quinoa helps preserve body weight under diabetic conditions, likely due to improved metabolic regulation. A similar weight-restorative effect of quinoa was previously reported in cisplatin-treated rats [28], reinforcing its potential in managing diabetes-associated cachexia.

### Effects on glycemic control

Quinoa supplementation significantly improved glycemic control in diabetic rats, especially in the D40 and D80 groups, compared to diabetic controls (D0). These findings are consistent with other studies demonstrating the antihyperglycemic effects of quinoa [29, 30], including models of fructose-induced metabolic dysfunction [31].

Mechanistically, quinoa’s hypoglycemic effects may be attributed to its high dietary fiber and complex carbohydrate content, which enhance insulin sensitivity and promote a gradual release of glucose [29]. Its inherently low glycemic index supports a slower rate of glucose absorption, thereby reducing postprandial glucose surges [4]. These properties are particularly advantageous in diabetic conditions characterized by impaired glycemic regulation.

At the molecular level, quinoa’s polyphenols, especially those in red quinoa, inhibit α-glucosidase – an enzyme responsible for carbohydrate digestion – thereby mitigating postprandial hyperglycemia [32]. In addition, phenolic compounds in quinoa have also been shown to inhibit α-amylase activity, offering a dual mechanism to suppress glucose absorption [33].

### Effects on lipid profile

Diabetic rats in this study exhibited elevated levels of triglycerides, total cholesterol, LDL, and VLDL cholesterol – hallmarks of diabetic dyslipidemia. This dyslipidemia is driven by excessive lipoprotein production and impaired clearance, secondary to insulin resistance, elevated free fatty acids, and chronic inflammation [34].

Quinoa supplementation resulted in significant reductions in VLDL cholesterol across all diabetic treatment groups (D20, D40, and D80) and an increase in HDL cholesterol in the D20 group. These observations align with previous reports in both animal and human studies, including those involving fructose-fed rats [31], young adults [16], and postmenopausal women [17].

Quinoa’s beneficial effects on lipid metabolism may be attributed to multiple bioactive compounds, including soluble fiber, saponins, and squalene [31, 35]. Soluble fiber enhances cholesterol excretion through bile acid binding, delays gastric emptying, and attenuates postprandial lipid responses [36]. Saponins are known to suppress hepatic cholesterol synthesis by downregulating hydroxy methylglutaryl coenzyme A reductase expression [35]. Moreover, quinoa’s unsaturated fatty acids may support HDL elevation and reverse cholesterol transport [37], contributing to a reduced risk of atherosclerosis [38, 39].

Differences in lipid outcomes across studies may be due to variability in quinoa preparation, dosage, and duration of administration. Processing methods influence the bioavailability of quinoa’s functional constituents, as demonstrated by studies comparing raw and thermally treated seeds [40].

### Hepatoprotective effects and safety profile

Our findings indicate a significant hepatoprotective effect of quinoa, evidenced by a 23.5% reduction (p < 0.01) in ALT levels among all quinoa-fed diabetic groups. These results are consistent with previous studies that demonstrated quinoa’s protective effects against hepatotoxicity, including carbon tetrachloride-induced liver injury [41], though not all studies report uniform outcomes [16].

The hepatoprotective mechanism likely involves quinoa’s polyphenolic constituents – kaempferol, quercetin, rutin, vanillic acid, and ferulic acid – which mitigate oxidative stress and hepatic inflammation [9]. Restoration of ALT levels to within normal ranges and the absence of histopathological abnormalities support the conclusion that quinoa enhances hepatic resilience under diabetic stress.

Furthermore, no adverse hematological or histo-logical effects were observed in either normal or dia-betic rats, underscoring quinoa’s short-term safety. Nevertheless, longer-term studies are needed to conf-irm these findings and assess potential morphological alterations.

### Modulation of intestinal function and gut health

Emerging evidence suggests that quinoa has a positive impact on gut health, which may contribute to its antidiabetic properties. During digestion, quinoa proteins generate peptides that inhibit enzymes that degrade incretins, potentially enhancing insulin activity [42]. In addition, fermentation of quinoa polysaccharides by gut microbiota increases short-chain fatty acid (SCFA) production, which in turn modulates glucose metabolism and energy homeostasis [43, 44].

Studies have shown that quinoa consumption improves glycemic and lipid profiles and increases SCFA secretion in obese diabetic mice, partly by correcting gut microbial imbalances [45]. Poorly absorbed saponins remain in the intestinal lumen, promoting microbial fermentation. Quinoa polyphenols also modulate digestive enzyme activity and gut flora composition. Its polysaccharides act as prebiotics, supporting the growth of *Bifidobacteria* and suppressing pathogenic organisms [46].

In murine models of colitis induced by dextran sodium sulfate, quinoa supplementation restored gut architecture and improved inflammatory outcomes [47]. These findings reinforce quinoa’s role in maintaining gut integrity and microbial balance.

### Selective therapeutic action and safety

A key observation of this study is quinoa’s selective therapeutic efficacy. While it had minimal effects in normal rats, quinoa significantly improved body weight, glucose control, and lipid parameters in diabetic rats. This selectivity, along with the absence of adverse hematological changes in either group, underscores quinoa’s promise as a targeted and safe intervention for diabetes-related metabolic dysfunctions.

## CONCLUSION

This study demonstrates the short-term therapeutic potential of quinoa supplementation in the management of diabetes-related metabolic disturbances. Supplementation with 40% and 80% quinoa diets significantly improved glycemic control, as evidenced by a 17% reduction in fasting blood glucose levels in diabetic rats (p < 0.05). In addition, all quinoa-fed diabetic groups exhibited reduced serum ALT levels (p < 0.01), indicating hepatoprotective effects. Improvements in lipid profiles were observed through decreased VLDL cholesterol and increased HDL cholesterol, while body weight loss in diabetic rats was attenuated in a dose-dependent manner. Notably, no adverse hematological or histopathological effects were observed in either diabetic or normal rats, reinforcing the safety of quinoa at high dietary concentrations over a 30-day period.

These findings support the potential inclusion of quinoa as a functional dietary adjunct in diabetes management protocols. Its favorable nutrient profile and bioactive compounds offer a non-pharmacological strategy to ameliorate hyperglycemia, dyslipidemia, and liver dysfunction – common comorbidities in diabetes. The absence of negative effects in healthy subjects further supports its applicability in both preventive and therapeutic dietary regimens.

A key strength of this study lies in its comparative evaluation of multiple quinoa concentrations (0%, 20%, 40%, and 80%) across both diabetic and non-diabetic models, enabling dose-response interpretation and safety assessment. The comprehensive analysis of metabolic, biochemical, hematological, and histological outcomes enhances the translational relevance of the findings.

Several limitations must be acknowledged. First, the 30-day duration may not fully capture long-term metabolic outcomes of quinoa supplementation. Second, while significant physiological benefits were observed, underlying molecular mechanisms were not investigated. Third, findings from this STZ-induced diabetic rat model may not directly extrapolate to human diabetic populations. Future research should include long-term studies, detailed molecular analyses, clinical trials, and evaluations of various quinoa cultivars and processing methods. In addition, measurement of baseline (pre-intervention) and post-intervention parameters would have strengthened the findings.

Further studies should explore the long-term efficacy and safety of quinoa supplementation, particularly in chronic diabetic models and human clinical trials. Investigation of underlying molecular mechanisms – including gene expression, insulin signaling pathways, and gut microbiota modulation – will offer deeper mechanistic insights. Evaluating the impact of different quinoa varieties and processing methods on bioactivity and bioavailability is also warranted to optimize therapeutic formulations.

Quinoa emerges as a promising, safe, and nutritionally beneficial dietary intervention for managing diabetes-related metabolic dysfunctions. Its multi-targeted effects, including glycemic regulation, lipid modulation, and liver protection, underscore its potential as a functional food ingredient in both clinical and public health settings aimed at mitigating the global diabetes burden.

## Data Availability

All the generated data are included in the manuscript.

## AUTHORS’ CONTRIBUTIONS

MA: Study design, sample collection, analysis and interpretation of data, manuscript drafting and revision. SDM: Study design, analysis and interpretation of data, and manuscript drafting and revision. DP: Analysis of data and manuscript revision. CMS and KV: Study design and manuscript revision. DU: Study design, statistical analysis and interpretation of data, and manuscript drafting and revision. NA and CS: Acquisition of data and manuscript revision. All authors have read and approved the final manuscript.
